# Early-onset colorectal cancer in Sweden: increase in distal tumors but a shift in right-sided incidence, 2020-2024

**DOI:** 10.1093/jncics/pkag097

**Published:** 2026-09-23

**Authors:** Shabane Barot, Cecilia Radkiewicz

**Affiliations:** Department of Clinical Science and Education, Karolinska Institutet, Stockholm, Sweden; Department of Oncology, Södersjukhuset, Stockholm, Sweden; Clinical Pharmacoepidemiology, Clinical Epidemiology Unit, Department of Medicine, Solna, Karolinska Institutet, Stockholm, Sweden; Department of Surgery and Oncology, Capio Sankt Görans Hospital, Stockholm, Sweden

## Abstract

Early-onset colorectal cancer (EOCRC; age <50 years) has increased in high-income countries. We updated a nationwide Swedish cohort study with data from 2020 to 2024 to compare recent trends with those reported during 1993-2019. Using the National Cancer Register, we identified all incident EOCRC at ages 20-49 years. Temporal trends in age-standardized incidence were assessed using segmented Poisson regression with a breakpoint at 2020 to estimate annual percentage changes (APCs). A total of 1616 EOCRC cases were diagnosed during 2020-2024. Annual percentage changes for left-sided colon and rectal cancers were consistent with continued increases in incidence. In contrast, right-sided colon cancer showed a significant change in trend (*P *= .01), with a nonsignificant decline during 2020-2024 (APC −3.48, 95% CI = −7.88 to 1.14). These findings indicate that the continued rise in EOCRC is primarily driven by distal tumors, that is, left-sided colon and rectal cancers. Continued surveillance is needed to clarify long-term patterns and underlying drivers.

Early-onset colorectal cancer (EOCRC; diagnosed before age 50 years) is an emerging global health concern. Although the overall incidence of colorectal cancer (CRC) has stabilized or declined in several high-income countries, EOCRC rates continue to rise, with the most consistent increases observed in high-income settings and in left-sided colon and rectal cancers, that is, distal tumors, compared with right-sided colon cancer, or proximal disease.^1^ A pronounced birth-cohort effect suggests that early-life environmental exposures drive this increase, though the underlying mechanisms remain unclear.^2^ During the same period, the prevalence of obesity among children and adolescents has increased markedly and has been proposed as a potential contributor to the rising EOCRC incidence.^3^ Supporting this hypothesis, population-based studies from the US have demonstrated rising incidence rates of multiple obesity-associated cancers, including CRC, in progressively younger birth cohorts, indicating increasing exposure to carcinogens over time.^4^ We previously reported an increasing incidence of EOCRC in Sweden during 1993-2019 in a nationwide study that included all incident CRC cases.^5^ Rising rates were observed across anatomical subsites, with annual percentage changes (APCs) of 2.64 (95% CI = 2.03 to 3.27) for right-sided colon cancer, 2.41 (95% CI = 1.83 to 2.99) for left-sided colon cancer, and 2.04 (95% CI = 1.51 to 2.56) for rectal cancer.^5^

To assess recent EOCRC incidence trends, we extended our analyses to include cases diagnosed from 2020 to 2024, using the same anatomical classifications and statistical approach as in our previous report.^5^ The Swedish National Cancer Register was used to identify all first-incident adenocarcinomas, classified according to ICD-O-2, in the right colon (C180, C182, C183, C184), left colon (C185, C186, C187, C19), and rectum (C20) among adults aged 20-49 years during this period. Appendix cancer (ICD-O-2 C181) was excluded due to its distinct biology and risk factor profile. Publicly available data on population counts by age and calendar year from Statistics Sweden were used as denominators for incidence estimation. Baseline characteristics were summarized using counts and proportions.

To assess whether temporal trends in age-standardized incidence rates (ASIRs) changed after 2019, we applied segmented Poisson regression models with a predefined breakpoint at 2020. The breakpoint was prespecified to evaluate whether the trends observed during 1993-2019 persisted beyond 2019. Annual ASIRs were calculated using direct standardization to the 2019 Swedish population, consistent with our previous analysis to ensure comparability, and expressed per 100 000 person-years. We then fitted Poisson regression models including calendar year (continuous) and a post-2020 spline term. This parameterization allowed estimation of separate APCs for the periods 1993-2019 and 2020-2024. Evidence of a change in trend was evaluated by testing the statistical significance of the post-2020 coefficient. For comparison, we fitted a data-driven piecewise regression model with an estimated rather than prespecified breakpoint. All statistical analyses were conducted using Stata version SE19.0 (StataCorp). The study was approved by the Swedish Ethical Review Authority (2026-00870-02; amendment covering updated data for 2020-2024).

A total of 1616 incident EOCRC cases were identified, with most occurring in the left-sided colon and rectum (Table 1). The majority of right-sided colon cancers (55.5%) and rectal (56.3%) cancers occurred in men, whereas the opposite pattern was observed for left-sided colon cancer (45.1% men). At diagnosis, between 23.0% (rectal) and 30.3% (left-sided colon) of EOCRC cases presented with synchronous metastases. Incidence trends for left-sided colon and rectal cancers during 2020-2024 were consistent with those reported for 1993-2019, with APCs of 1.64 (95% CI = −2.51 to 5.96) and 1.97 (95% CI = −1.84 to 5.92), respectively (Figure 1). In contrast, a statistically significant change in trend was observed for right-sided colon cancer (P for change in trend = 0.01), with a non-significant decline during 2020-2024 (APC −3.48, 95% CI = −7.88 to 1.14) compared with 1993-2019. Given the limited number of cases in 2020-2024, the APC estimates should be interpreted primarily in terms of direction rather than statistical significance, as short-term fluctuations may have a relatively large influence on trend estimates. The data-driven model did not confirm a distinct breakpoint for right-sided colon cancer, suggesting that the apparent post-2019 change identified in the prespecified analysis should be interpreted cautiously and requires longer follow-up before firm conclusions can be drawn. Minor differences in the pre-2020 slope estimates relative to our original report are expected, as parameters were re-estimated within a segmented regression framework that jointly models a post-2020 change in trend.

**Figure 1.**
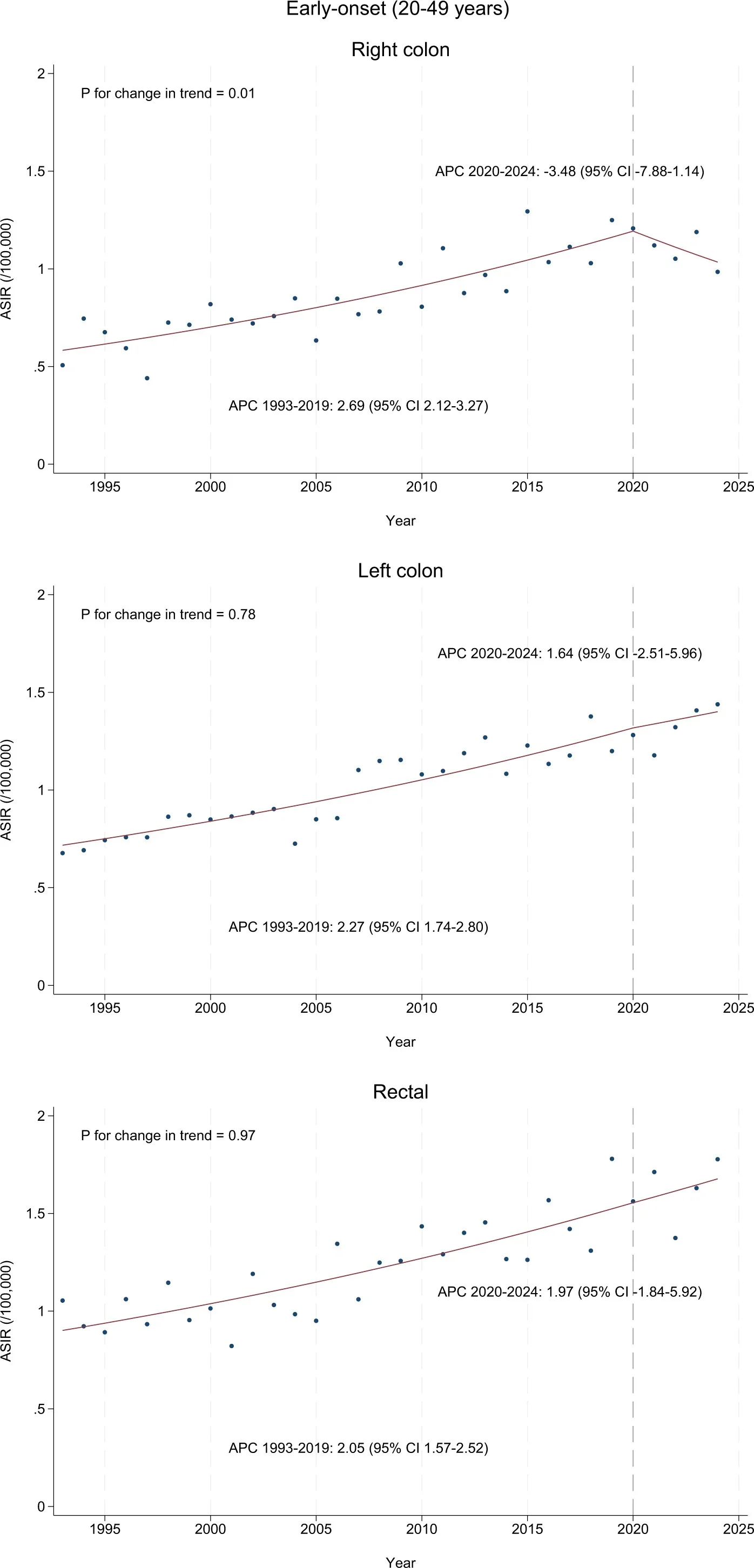
Age-standardized incidence rates (ASIRs) of early-onset (20-49 years) colorectal cancer per 100 000 person-years, 1993-2024. Dots indicate observed estimates, and solid lines represent fitted trends from segmented Poisson regression with a breakpoint at 2020. Annual percentage changes (APCs) and 95% CIs are reported for 1993-2019 and 2020-2024. *P* values test for a change in trend at 2020.

**Table 1:** Clinicopathological characteristics of early-onset (age 20-49 years) and later-onset (age 50-99 years) colorectal adenocarcinoma.

			Right colon		Left colon		Rectal	
			Early-onset		Early-onset		Early-onset	
Total:			*n*	%	*n*	%	*n*	%
	1616	(100%)						
			445	27.5	528	32.7	643	39.8
**Sex:**								
		Men	247	55.5	238	45.1	362	56.3
		Women	198	44.5	290	54.9	281	43.7
**Year:**								
		2020	96	21.6	102	19.3	124	19.3
		2021	90	20.2	94	17.8	136	21.2
		2022	85	19.1	105	19.9	110	17.1
		2023	96	21.6	112	21.2	131	20.4
		2024	78	17.5	115	21.8	142	22.1
**Adenocarcinoma:**								
		Classic	383	86.1	497	94.1	613	95.3
		Mucinous	53	11.9	24	4.5	26	4.0
		Undifferentiated	9	2.0	7	1.3	4	0.6
**Stage:**								
		M0	301	67.6	346	65.5	490	76.2
		M+	124	27.9	160	30.3	148	23.0
		MX	20	4.5	22	4.2	5	0.8

Abbreviations: M0 = non-metastatic disease; M+ = metastatic disease; MX = metastatic stage missing. Data are presented as numbers (*n*) and percentages (%).

Globally, the rise in EOCRC has largely been driven by tumors of the left colon and rectum. A nationwide study of CRC incidence trends in Norway during 1993-2022 reported increases in left-sided colon and rectal cancer similar to those observed in our previous study, whereas the incidence of proximal or right-sided colon cancer remained stable over the same period.^6^

Right-sided colon cancer may differ from left-sided colon and rectal cancer in risk factor profiles: hereditary predisposition and older age are more strongly associated with right-sided cancers, whereas certain lifestyle-related exposures, such as obesity, unhealthy diets, and physical inactivity, appear to play a greater role in left-sided colon and rectal cancers.^3^ However, the mechanisms underlying colorectal carcinogenesis overlap across anatomical subsites.^7^

In the absence of population-based screening among individuals younger than 50 years in Sweden, changes in healthcare utilization and diagnostic practices may nevertheless have influenced case detection over time.

The increase in right-sided EOCRC observed from 1993 to 2019 may partly reflect the implementation of nationwide surveillance programs for individuals with familial CRC and hereditary syndromes, including Lynch syndrome, introduced in Sweden in the 1990s. Although 5%-9% of EOCRC cases are attributable to Lynch syndrome, the absolute number of cancers detected through genetic testing and surveillance remains modest and is unlikely to explain population-level trends.^8^ The observed decline in right-sided EOCRC incidence during 2020-2024 may instead reflect disruptions in healthcare delivery during the COVID-19 pandemic, potentially affecting patients with more nonspecific symptoms, as is typical for right-sided tumors.^9^ However, the proportion of patients presenting with metastatic disease during 2020-2024 was similar to that observed before the pandemic period, providing little support for substantial diagnostic delays. Additional data are needed to clarify the potential impact of COVID-19-related disruptions on CRC diagnostics.

Notably, we observed no corresponding decline in left-sided colon and rectal cancers, which account for the majority of EOCRC cases. These findings align with global trends showing a continued increase in EOCRC, driven primarily by cancers more closely linked to modifiable risk factors. The persistence of these patterns in the most recent calendar period suggests that the rise in EOCRC is unlikely to be transient. This underscores the need for urgent efforts to elucidate the underlying causes and to ensure that healthcare systems are prepared to manage a growing population of younger patients with CRC. Without targeted preventive strategies and increased awareness, EOCRC is likely to remain a sustained and expanding public health challenge.

## Data Availability

Aggregated data supporting study findings are available from the corresponding author upon reasonable request. Individual-level data cannot be shared due to legal and ethical restrictions, including the General Data Protection Regulation (GDPR). Researchers may access the data upon appropriate ethical approval by applying to the Swedish National Board of Health and Welfare: https://www.socialstyrelsen.se/en/statistics-and-data/questions-about-registers-statistics-and-data-requests/.
